# Supplementary material for: The Evolution and Characterization of the RNA Interference Pathways in Lophotrochozoa
Source: Genome Biol Evol. 2024 May 7;16(5):evae098. doi: 10.1093/gbe/evae098 (PMC11114477; doi:10.1093/gbe/evae098)
Supplement: evae098_Supplementary_Data [file evae098_supplementary_data.zip › Supplementary_materials.zip!/selected_miRNAs/NoGe_selected_miRNAs/mirdeep_pdf_files/NMRB01000177.1_Notospermus_geniculatus_strain_Ushimado_scaffold177,_whole_genome_shotgun_sequence_6428.pdf]

Provisional ID : NMRB01000177.1_Notospermus_geniculatus_strain_Ushimado_scaffold177,_whole_genome_shotgun_sequence_6428
Score total : 9801
Score for star read(s) : 3.9
Score for read counts : 9792.3
Score for mfe : 1.7
Score for randfold : 0
Score for cons. seed : 3
Total read count : 19219
Mature read count : 19081
Loop read count : 0
Star read count : 138

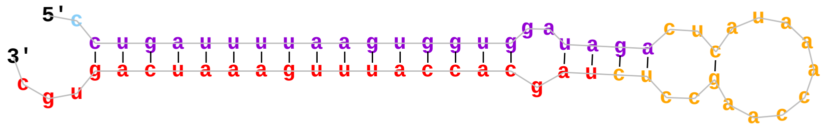

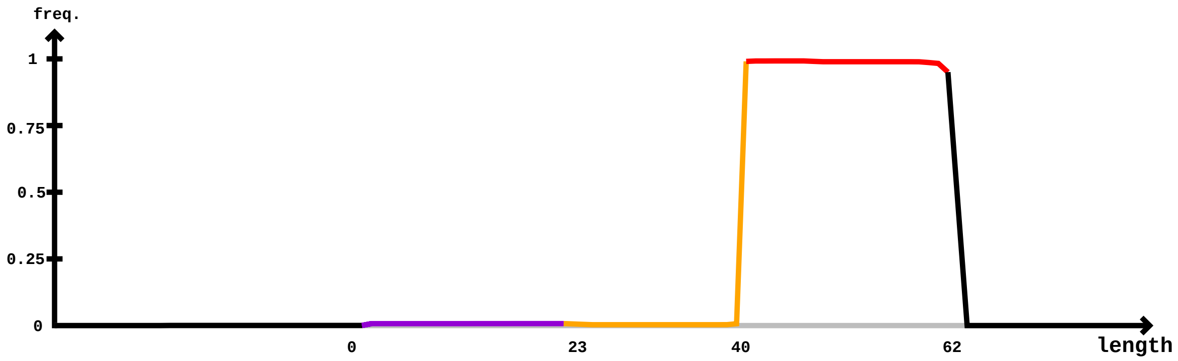

Star Mature

```
5'- gaugaugccacagaauauggacccgaggagccugauuuuaagugguggauagacucauaaaccaagccucuagcaccauuugaaaucagugcugcugggcguugcuagucga  -3'  obs
    gaugaugccacagaauauggacccgaggagccugauuuuaagugguggauagacucauaaaccaagccucuagcaccauuugaaaucagugcugcugggcguugcuagucga        exp
    ...........((.((..(((((.((.((((((((((((((((((..((((..(.........)..)))).))))))))))))))).))).)).)).))).)).)).. reads  mm  sample
    ..........cagaauauggacccgaggagc.................................................................................  2  0  Mm1
    ......................................................................uagcaccauuugaaaucagu......................  3  0  Mm1
    ......................................................................uagcaccauuugaaaucagug.....................  137  0  Mm1
    ......................................................................uagcaccauuugaaauAagug.....................  1  1  Mm1
    ......................................................................uagcaccauuugaaaucUgug.....................  1  1  Mm1
    ......................................................................uGgcaccauuugaaaucagugc....................  88  1  Mm1
    ......................................................................uagcaccauuugaaaucagugc....................  24  0  Mm1
    ......................................................................uagcaccauuugaaaucCgugc....................  1  1  Mm1
    ......................................................................uagcaccauuugaaaucaguUcu...................  3  1  Mm1
    ......................................................................uGgcaccauuugaaaucagugcu...................  1  1  Mm1
    ......................................................................uagcaccauuugaaaucagugcA...................  10  1  Mm1

    ..........cagaauauggacccgaggagc.................................................................................  4  0  Mm2
    ..........................................ugguggauagacucauaaaccaagccu...........................................  2  0  Mm2
    ..............................................ggauagacucauaaaccaagccu...........................................  1  0  Mm2
    .....................................................cucauaaaccaagccucuGgcacc...................................  1  1  Mm2
    .....................................................cucauaaaccaagccucuGgcacca..................................  1  1  Mm2
    ......................................................ucauaaaccaagccucuGgcacca..................................  1  1  Mm2
    ....................................................................cuGgcaccauuugaaaucagug.....................  3  1  Mm2
    ....................................................................cuGgcaccauuugaaaucagugc....................  1  1  Mm2
    ......................................................................uagcaccauuugaaaucagu......................  1  0  Mm2
    ......................................................................uGgcaccauuugaaaucagu......................  3  1  Mm2
    ......................................................................uagcaccauuugaaaucagug.....................  19  0  Mm2
    ......................................................................uGgcaccauuugaaaucagug.....................  46  1  Mm2
    ......................................................................uCgcaccauuugaaaucagug.....................  1  1  Mm2
    ......................................................................uagcaccauuugaaaucagugc....................  450  0  Mm2
    ......................................................................uagcGccauuugaaaucagugc....................  5  1  Mm2
    ......................................................................uagcaccauuugaaauAagugc....................  4  1  Mm2
    ......................................................................uagcaccauuugaaaucCgugc....................  1  1  Mm2
    ......................................................................uagcaccauuugaaaucagugG....................  7  1  Mm2
    ......................................................................uagcaccauuugaaaucGgugc....................  3  1  Mm2
    ......................................................................uagcaccauuugaaaucaguAc....................  1  1  Mm2
    ......................................................................uagcaccauuAgaaaucagugc....................  2  1  Mm2
```

Star **Mature**

gaugaugccacagaauauggacccgaggagccugauuuuaagugguggauagacucauaaaccaagccucuagcaccauuugaaaucagugcugcugggcguugcuagucga			
....uagcaccauuugaaauUagugc....................	2	1	Mm2
....uagcaccauuugaaaucagCgc....................	1	1	Mm2
....uagcaccauuugaaauGagugc....................	2	1	Mm2
....uGgcaccauuugaaaucagugc....................	1118	1	Mm2
....uagcaccauuugaaaucaAugc....................	1	1	Mm2
....uagcaccauuugaaaucUgugc....................	1	1	Mm2
....uCgcaccauuugaaaucagugc....................	1	1	Mm2
....Nagcaccauuugaaaucagugc....................	1	1	Mm2
....uagcaccauuugaaaucaguUcu...................	17	1	Mm2
....uagcaccauuugaaaucagugcA...................	1	1	Mm2
....uGgcaccauuugaaaucagugcu...................	3	1	Mm2
....Ggcaccauuugaaaucagugc....................	1	1	Mm2
....ugguggauagacucauaaaccaagccu...	1	0	Mm3
....ucuGgcaccauuugaaaucagug.....................	1	1	Mm3
....cuGgcaccauuugaaaucagug.....................	5	1	Mm3
....cuagcaccauuugaaaucagugc....................	1	0	Mm3
....cuGgcaccauuugaaaucagugc....................	1	1	Mm3
....uGgcaccauuugaaaucagu......................	7	1	Mm3
....uagcaccauuugaaaucagu......................	16	0	Mm3
....uagcaccauuugaaaucagug.....................	20	0	Mm3
....uagcaccauuugaaauUagug.....................	1	1	Mm3
....uGgcaccauuugaaaucagug.....................	25	1	Mm3
....uagcaccauuGgaaaucagugc....................	1	1	Mm3
....uagcaccauuugaaaucGgugc....................	1	1	Mm3
....Cagcaccauuugaaaucagugc....................	1	1	Mm3
....uagcaccauuAgaaaucagugc....................	1	1	Mm3
....uUgcaccauuugaaaucagugc....................	1	1	Mm3
....uGgcaccauuugaaaucagugc....................	334	1	Mm3
....uagcaccauuugaaaucagugc....................	204	0	Mm3
....uagcaccauuugaaauGagugc....................	1	1	Mm3
....uagcaccauuugaaaucagugcA...................	2	1	Mm3
....uagcaccauuugaaaucaguUcu...................	28	1	Mm3
....agcaccauuugaaaucagug.....................	1	0	Mm3
....caccauuugaaaucagugc....................	1	0	Mm3
..........cagaauauggacccgaggagc...	1	0	Fm1
....ucauaaaccaagccucuGgcacca..................................	10	1	Fm1
....cuGgcaccauuugaaaucagugc....................	9	1	Fm1
....uagcaccauuugaaauca........................	4	0	Fm1
....uGgcaccauuugaaaucagug.....................	32	1	Fm1
....uagcaccauuugaaaucagug.....................	4	0	Fm1
....uagcaccauuugaaaucagugA....................	2	1	Fm1
....uagcaccauuugaaauUagugc....................	4	1	Fm1
....uagcaccauuugaCaucagugc....................	1	1	Fm1
....uagcaccauuugaaaucUgugc....................	13	1	Fm1
....uagcaccauuugaGaucagugc....................	2	1	Fm1
....uagcaccauuCgaaaucagugc....................	2	1	Fm1
....uagcaccauuAgaaaucagugc....................	6	1	Fm1
....uagcaccauuGgaaaucagugc....................	4	1	Fm1
....uaUcaccauuugaaaucagugc....................	2	1	Fm1
....uagcaUcauuugaaaucagugc....................	2	1	Fm1
....uagcaccauuugaaaucagugG....................	43	1	Fm1
....uGgcaccauuugaaaucagugc....................	9688	1	Fm1
....uagcacAauuugaaaucagugc....................	1	1	Fm1
....uagcacUauuugaaaucagugc....................	2	1	Fm1
....uagcaccUuuugaaaucagugc....................	1	1	Fm1
....uagcaccauuugaaaucCgugc....................	15	1	Fm1
....uagcaccauuugaaauAagugc....................	20	1	Fm1
....uagcaccauuugaaaucagugU....................	2	1	Fm1
....uagcaccauuugaaauGagugc....................	2	1	Fm1
....uagcGccauuugaaaucagugc....................	6	1	Fm1
....uagcaccauuugGaaucagugc....................	1	1	Fm1
....uUgcaccauuugaaaucagugc....................	3	1	Fm1
....uagcaccauuugaaaucaUugc....................	1	1	Fm1
....Nagcaccauuugaaaucagugc....................	1	1	Fm1
....uagcaccauuugaaaucagugc....................	3148	0	Fm1
....uagcaccauuugaaaucGgugc....................	10	1	Fm1
....uagcaGcauuugaaaucagugc....................	1	1	Fm1
....uagcaccauuugaaaucagugcu...................	11	0	Fm1

Star **Mature**

```
gaugaugccacagaauauggacccgaggagccugauuuuaagugguggauagacucauaaaccaagccucuagcaccauuugaaaucagugcugcugggcguugcuagucga
......................................................................uGgcaccauuugaaaucagugcu...................   1     1   Fm1
......................................................................uagcaccauuugaaaucagugcA...................   3     1   Fm1
......................................................................uagcaccauuugaaaucaguUcu...................   1     1   Fm1
.......................................................................agcaccauuugaaaucagugc....................   8     0   Fm1
.......................................................................Ggcaccauuugaaaucagugc....................   13    1   Fm1

...............................cugauuuuaagugguggauaga...........................................................   134   0   Fm2
...............................cugauuuuaagugguggGuaga...........................................................   1     1   Fm2
...............................cugauuCuaagugguggauaga...........................................................   1     1   Fm2
...............................cugauuuuaaguggugUauaga...........................................................   1     1   Fm2
...............................cugauuuuaagugguggUuaga...........................................................   1     1   Fm2
......................................................ucauaaaccaagccucuGgcacc...................................   29    1   Fm2
......................................................ucauaaaccaagccucuGgcacca..................................   16    1   Fm2
.....................................................................cuGgcaccauuugaaaucagug.....................   19    1   Fm2
.....................................................................cuGgcaccauuugaaaucagugc....................   19    1   Fm2
......................................................................uGgcaccauuugaaaucagug.....................   13    1   Fm2
......................................................................uagcaccauuugaaauGagug.....................   1     1   Fm2
......................................................................uagcaccauuugaaaucagug.....................   14    0   Fm2
......................................................................uagcaccauuugaaauAagugc....................   3     1   Fm2
......................................................................uagcaccauuugaaaucagugA....................   1     1   Fm2
......................................................................uGgcaccauuugaaaucagugc....................   726   1   Fm2
......................................................................uagcaccauuugaaaucCgugc....................   1     1   Fm2
......................................................................Aagcaccauuugaaaucagugc....................   1     1   Fm2
......................................................................uUgcaccauuugaaaucagugc....................   1     1   Fm2
......................................................................uagcaccauuugaaauUagugc....................   1     1   Fm2
......................................................................uCgcaccauuugaaaucagugc....................   2     1   Fm2
......................................................................uagcaccauuugaaaucagugc....................   248   0   Fm2
......................................................................uagcaccauuugaaaucagugG....................   4     1   Fm2
......................................................................uagcaccauuugaaaucaguUcu...................   9     1   Fm2
......................................................................uagcaccauuugaaaucagugcA...................   10    1   Fm2

....................................................................ucuGgcaccauuugaaaucagug.....................   2     1   Fm3
.....................................................................cuGgcaccauuugaaaucagug.....................   8     1   Fm3
.....................................................................cuGgcaccauuugaaaucagugc....................   4     1   Fm3
......................................................................uagcaccauuugaaaucag.......................   52    0   Fm3
......................................................................uagcaccauuugaaaucaU.......................   1     1   Fm3
......................................................................uaUcaccauuugaaaucagu......................   4     1   Fm3
......................................................................uagcaccauuugaaaucagu......................   27    0   Fm3
......................................................................uGgcaccauuugaaaucagu......................   1     1   Fm3
......................................................................uagcaccauuuAaaaucagu......................   1     1   Fm3
......................................................................uagcaccauuugaaaucCgug.....................   1     1   Fm3
......................................................................uagcaccauuugaaaucagug.....................   210   0   Fm3
......................................................................uagcaccauuugaaaucagCg.....................   1     1   Fm3
......................................................................uGgcaccauuugaaaucagug.....................   55    1   Fm3
......................................................................uagcaccauuugaaaucaguA.....................   1     1   Fm3
......................................................................uagcaccauuugaaauUagug.....................   2     1   Fm3
......................................................................uagcaccauuugaaaucGgug.....................   3     1   Fm3
......................................................................uagcaccauuugaGaucagug.....................   1     1   Fm3
......................................................................uagcaccauuugaaauAagug.....................   2     1   Fm3
......................................................................uagcaccauuAgaaaucagug.....................   2     1   Fm3
......................................................................uagcaccauuAgaaaucagugc....................   1     1   Fm3
......................................................................uagcaccauuCgaaaucagugc....................   3     1   Fm3
......................................................................uagcaccauuugaaaucCgugc....................   3     1   Fm3
......................................................................uagcaccauuugaaauAagugc....................   4     1   Fm3
......................................................................uagcaccauuugaaauUagugc....................   1     1   Fm3
......................................................................uagcaccauuugaaaucaAugc....................   1     1   Fm3
......................................................................uagcaccauuGgaaaucagugc....................   2     1   Fm3
......................................................................uagcaccauuugaaaucagugc....................   587   0   Fm3
......................................................................uagcaccauuugaaauGagugc....................   2     1   Fm3
......................................................................uagcaccauuugaaaucagugA....................   3     1   Fm3
......................................................................uagcaccauuugaaaucagugU....................   7     1   Fm3
......................................................................Cagcaccauuugaaaucagugc....................   1     1   Fm3
......................................................................uagcaccauuugaaaucUgugc....................   3     1   Fm3
......................................................................uagcaccauuugaaaucGgugc....................   1     1   Fm3
......................................................................uagcaccauuugaaaucaguAc....................   1     1   Fm3
......................................................................uagcGccauuugaaaucagugc....................   1     1   Fm3
......................................................................uGgcaccauuugaaaucagugc....................   1300  1   Fm3
......................................................................uGgcaccauuugaaaucagugcu...................   3     1   Fm3
......................................................................uagcaccauuugaaaucagugcu...................   2     0   Fm3
......................................................................uagcaccauuugaaaucaguUcu...................   8     1   Fm3
```

```
                              Star                                    Mature
gaugaugccacagaauauggacccgaggagccugauuuuaagugguggauagacucauaaaccaagccucuagcaccauuugaaaucagugcugcugggcguugcuagucga
.......................................................................agcaccauuugaaaucagug.....................	1	0	Fm3
.......................................................................Ggcaccauuugaaaucagugc....................	3	1	Fm3
.......................................................................agcaccauuugaaaucagugc....................	2	0	Fm3
.........................................................................caccauuugaaaucagugcu...................	3	0	Fm3
```
